# Reply to: The dark side of T2: central nervous system lesions with low signal intensity on T2-weighted imaging

**DOI:** 10.1590/0100-3984.2024.0087

**Published:** 2025-02-07

**Authors:** Venkatraman Indiran

**Affiliations:** Department of Radiodiagnosis, Sree Balaji Medical College and Hospital, Chennai, Tamil Nadu, India. Email: ivraman31@gmail.com

Dear Editor,

I read with interest the excellent and exhaustive article describing the lesions showing T2 hypointense signal (T2 shortening) in the brain by Carpentieri-Primo et al.^(1)^. I would like to add one more pathology—non-ketotic hyperglycemia (NKH)—that can present with T2 hypointense signal. Uncontrolled NKH with or without significant serum osmolality abnormality can present with varied symptoms such as seizures, focal neurological deficit, movement disorders (unilateral hemichorea—hemiballism), hyperthermia and vestibular dysfunction^(2)^. NKH patients presenting with seizures show specific findings such as subcortical T2 hypointensity with other associated features such as overlying cortical T2 hyperintensity, focal cortical enhancement and bilateral T2 striatal hyperintensity^(2^, ^3)^. Possible mechanisms for subcortical T2 shortening/hypointensity include mineral deposition, free radical and iron accumulation and ischemia^(3^, ^4)^. Parietal and occipital lobes, the most commonly involved regions in NKH, show subcortical T2 and T2 FLAIR hypointense signal and mild hypointense signal on susceptibility weighted images, with or without overlying cortical restricted diffusion and contrast enhancement^(4)^. NKH patients presenting with hemichorea-hemiballism show T1 hyperintense and variable (iso/hypointense) T2 signal in basal ganglia/thalamus with gemistrocyte accumulation, hyperviscosity, haemorrhage, neuronal dysfunction and possible cytotoxic edema being the possible causes of the signal change^(3^, ^4^, ^5)^. Neonatal hypoglycemia is another condition that may sometimes show T2 shortening, albeit involving the cortex with T2 hyperintense subcortical white matter^(3)^. It is ideal to keep NKH as the possible diagnosis in a patient with seizure, uncontrolled hyperglycemia, and absence of ketones presenting with subcortical T2 hypointensity, cortical hyperintensity, and restricted diffusion on MRI to initiate prompt treatment.

## References

[1] Carpentieri-Primo P, Nahoum L, Almeida L (2024). The dark side of T2: central nervous system lesions with low
signal intensity on T2-weighted imaging. Radiol Bras.

[2] Panneer SB, Jain A (2024). Neuroimaging in uncontrolled hyperglycemia: a case series and
literature review. Egypt J Radiol Nucl Med.

[3] Bathla G, Policeni B, Agarwal A (2014). Neuroimaging in patients with abnormal blood glucose
levels. AJNR Am J Neuroradiol.

[4] Hiremath SB, Gautam AA, George PJ (2019). Hyperglycemia-induced seizures — understanding the
clinico-radiological association. Indian J Radiol Imaging.

[5] Hansford BG, Albert D, Yang E (2013). Classic neuroimaging findings of nonketotic hyperglycemia on
computed tomography and magnetic resonance imaging with absence of typical
movement disorder symptoms (hemichorea-hemiballism). J Radiol Case Rep.

